# Circadian Forced Desynchrony of the Master Clock Leads to Phenotypic Manifestation of Depression in Rats

**DOI:** 10.1523/ENEURO.0237-16.2016

**Published:** 2017-01-06

**Authors:** Miriam Ben-Hamo, Tracy A. Larson, Leanne S. Duge, Carl Sikkema, Charles W. Wilkinson, Horacio O. de la Iglesia, Mónica M. C. González

**Affiliations:** 1 Department of Biology; 2 Program in Neuroscience, University of Washington, Seattle, WA 98195; 3 Geriatric Research, Education and Clinical Center, VA Puget Sound Health Care System, Seattle, WA 98108; 4 Department of Psychiatry and Behavioral Sciences, University of Washington, Seattle, WA 98195

**Keywords:** behavior, circadian rhythms, depression, emotion, rat

## Abstract

In mammals, a master circadian clock within the suprachiasmatic nucleus (SCN) of the hypothalamus maintains the phase coherence among a wide array of behavioral and physiological circadian rhythms. Affective disorders are typically associated with disruption of this fine-tuned “internal synchronization,” but whether this internal misalignment is part of the physiopathology of mood disorders is not clear. To date, depressive-like behavior in animal models has been induced by methods that fail to specifically target the SCN regulation of internal synchronization as the mode to generate depression. In the rat, exposure to a 22-h light-dark cycle (LD22) leads to the uncoupling of two distinct populations of neuronal oscillators within the SCN. This genetically, neurally, and pharmacologically intact animal model represents a unique opportunity to assess the effect of a systematic challenge to the central circadian pacemaker on phenotypic manifestations of mood disorders. We show that LD22 circadian forced desynchrony in rats induces depressive-like phenotypes including anhedonia, sexual dysfunction, and increased immobility in the forced swim test (FST), as well as changes in the levels and turnover rates of monoamines within the prefrontal cortex. Desynchronized rats show increased FST immobility during the dark (active) phase but decreased immobility during the light (rest) phase, suggesting a decrease in the amplitude of the normal daily oscillation in this behavioral manifestation of depression. Our results support the notion that the prolonged internal misalignment of circadian rhythms induced by environmental challenge to the central circadian pacemaker may constitute part of the etiology of depression.

## Visual Abstract

**Figure:**
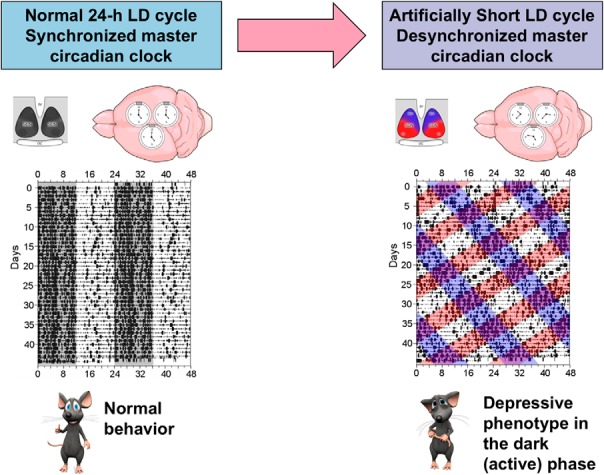

## Significance Statement

Previous studies hypothesized that disruption of the internal coordination of circadian rhythms by the master circadian clock may contribute to the etiology of mood disorders. This hypothesis has not yet been tested in genetically and neurologically intact animal models with impairment of the master clock’s ability to maintain circadian internal synchronization. We compared rats exposed to 22-h light-dark cycle, which leads to forced desynchrony of neuronal oscillators within the suprachiasmatic nucleus, to rats exposed to a normal 24-h light-dark cycle. We show that forced desynchrony induces a depressive phenotype that is particularly manifested during the animal's dark (active) phase. Our results demonstrate that prolonged internal misalignment of circadian rhythms may constitute part of the etiology of depression.

## Introduction

In mammals, circadian rhythms are coordinated by a master circadian clock located in the suprachiasmatic nucleus (SCN), where transcriptional and translational feedback loops within single cells oscillate with a period of approximately 24 h; these cells compose a neuronal network that generates robust endogenous circadian oscillations (Colwell, 2011). Direct retinal projections to the SCN via the retinohypothalamic tract (RHT) assure entrainment of the master clock—as well as the body rhythms it regulates—to the light-dark (LD) cycle (Golombek and Rosenstein, 2010). In this manner, the SCN governs a wide array of behavioral and physiological rhythms including the sleep-wake cycle, body temperature, hormone release, appetite, and cognitive functions (Saper et al., 2005). This regulation is achieved directly or through inputs to both central and peripheral extra-SCN circadian oscillators that locally time tissue-specific processes (Mohawk et al., 2012). Thus, the SCN master clock is responsible for maintaining phase coherence within the complex network of body oscillators and the rhythms they regulate. Disruption of this fine-tuned “internal synchronization” is often associated with both somatic and neuropsychiatric disorders (Takahashi et al., 2008; Wulff et al., 2010; McCarthy and Welsh, 2012; Morris et al., 2012; McClung, 2013).

Numerous lines of evidence support the association between abnormal endogenous circadian rhythms and affective disorders. Patients with affective disorders show disruptions in an array of circadian rhythms including sleep, body temperature rhythm, and hormone release (Boivin, 2000; Wirz-Justice, 2006), suggesting that mood disorders are linked to impairment of the master regulation of circadian rhythms rather than the disruption of isolated rhythmic outputs. Moreover, bright light therapy, sleep deprivation, sleep phase advance, and social rhythm therapy, which are all capable of resetting the phase of the master circadian clock, were shown to be effective in the treatment of affective disorders (Riemann et al., 2002; Frank et al., 2005; Golden et al., 2005; Hemmeter et al., 2010). In addition, the SCN projects directly or through relays to the prefrontal cortex, hippocampus, and amygdala (Leak and Moore, 2001) and trans-synaptically drives the circadian cellular activity of monoaminergic brain regions (Aston-Jones et al., 2001; Luo and Aston‐Jones, 2009), all of which are associated with mood regulation. Accordingly, exposure to short photoperiods or constant light can lead to behavioral symptoms of depression in different rodent models (Prendergast and Nelson, 2005; McClung, 2011), and rats exposed to constant darkness for several weeks show apoptosis in mood-related monoaminergic neurons in association with a depressive behavioral phenotype (Gonzalez and Aston-Jones, 2006, 2008). These environmental light effects were attributed in part to the master circadian clock. Finally, mice with mutated or knocked-down clock genes typically show abnormal affective behavior (Prickaerts et al., 2006; Roybal et al., 2007; Keers et al., 2012). These behavioral effects emerge, at least in part, from the absence of a functional clock within SCN neurons, as region-specific knockdown of the clock gene *Bmal1* in the SCN is associated with similar behavioral manifestations of depression (Landgraf et al., 2016).

Taken together, these findings have led to the hypothesis that the disruption of the circadian system and specifically the internal phase coordination of circadian rhythms by the master circadian clock may constitute part of the etiology of mood disorders. Nevertheless, this hypothesis has not yet been tested in genetically and neurologically intact animal models—better concurring with the etiology of affective disorders in humans—with targeted impairment of the master clock’s ability to maintain circadian internal synchronization. de la Iglesia et al. (2004) developed a genetically, neurally and pharmacologically intact rat model of “forced desynchrony,” in which the mere exposure to a symmetric 22-h light-dark cycle (LD22) leads to the uncoupling of two distinct populations of neuronal oscillators within the SCN: the ventrolateral SCN (vlSCN) and the dorsomedial SCN (dmSCN). Forced desynchronized rats present key properties of human forced desynchrony such as the desynchronized circadian regulation of temperature, sleep stages, melatonin, and cortisol (Cambras et al., 2007; Lee et al., 2009; Schwartz et al., 2009; Wotus et al., 2013). The purpose of the present study was to take advantage of the forced desynchronized rat to test the prediction that a systematic challenge to the central circadian pacemaker’s ability to maintain the internal synchronization of circadian rhythms will itself cause an altered affective state. We show that forced desynchrony of circadian rhythms in rats induces signature symptoms that characterize depression in animal models including anhedonia, sexual dysfunction, and increased immobility in the forced swim test (FST), as well as changes in the levels and turnover rates of monoamines within the prefrontal cortex (PFC).

## Materials and Methods

### Animals and housing

Adult Wistar male rats (2 months old, Charles River Laboratories, Raleigh, NC) were housed in individual transparent cages with *ad libitum* access to standard rodent diet (5001, LabDiet, St. Louis, MO) and water. Animals were kept in a ventilated chamber for 9–12 weeks at constant humidity and temperature (21 ± 1°C) under a 12:12 LD (12 h light, 12 h dark) cycle (control group, LD24) or under an 11:11 LD (11 h light, 11 h dark) cycle (desynchronized group, LD22). Light intensity, as measured inside the individual cages at the level of the rat’s eyes, was 64 ± 11 lux during the light phase and <1 lux dim red light during the dark phase. Animals were individually housed because group housing interferes with desynchrony and the recording of locomotor activity rhythm of each individual. In an effort to minimize stress during the behavioral tests, rats were briefly handled two to four times a week during the dark phase by the same experimenter that performed the behavioral tests. Females were not used for behavioral tests (except for testing male sexual behavior) because the interaction between circadian desynchrony and estrous cycle, which is expected to affect behavioral tests, would require more animals and a different experimental design. The Institutional Animal Care and Use Committee of the University of Washington approved all procedures.

### Forced desynchrony

**Figure 1.**
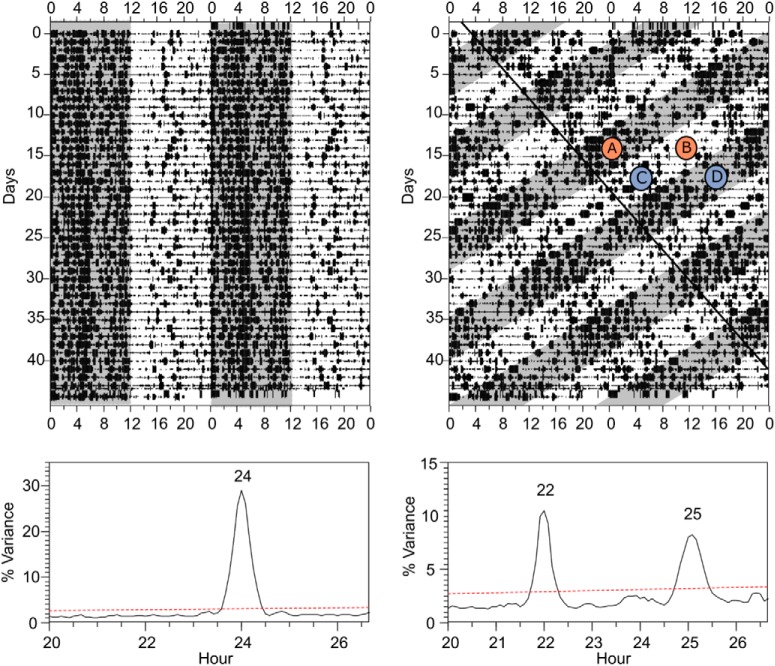
Forced desynchrony of locomotor activity. ***Top***, Double-plotted actograms for locomotor activity of representative rats maintained on symmetric 24-h (left; 12 h light, 12 h dark) or 22-h (right; 11 h light, 11 h dark) LD cycles. The gray shading indicates lights off. The black line on the LD22 plot indicates the onset of locomotor activity for the LD-dissociated bout of activity. Letters represent phases at which LD22 desynchronized animals were tested. During aligned days, animals were tested in the dark phase (***A***) or the light phase (***B***). During misaligned days, animals were also tested in the light phase (***C***) or the dark phase (***D***). Although all four phases are shown schematically in the same animal, each animal was tested only once at one of these four phases. ***Bottom***, Periodograms of the time series represented in each actogram. Analysis revealed a single statistically significant peak for the LD24 animal and two statistically significant peaks for the LD22 animal. The numbers above the peaks indicate the period of the significant peaks in hours.

Desynchrony was assessed by continuous recording of locomotor activity rhythms through crossed infrared beams and analysis with the Sokolove and Bushell (1978) periodogram. Infrared beam breaks were recorded in 10-min bins using Clocklab (Actimetrics, Wilmette, IL). After 3 weeks under LD22, two locomotor rhythms emerged with different period lengths, confirmed by Sokolove–Bushel periodogram analysis using El Temps (A. Díez-Noguera; Universidad de Barcelona, Barcelona, Spain; Fig. 1). One rhythm was entrained by the LD cycle and had a period of 22 h and the other was dissociated from the LD cycle and had a period >24 h (∼25 h). Rats that failed to desynchronize (∼20%) were removed from the study. The two locomotor activity rhythms of desynchronized rats periodically come in phase (aligned days) when the 22- and ∼25-h locomotor activity bouts overlap with each other (Fig. 1; circled A and B), and come out of phase (misaligned days), when one locomotor activity bout starts where the other ends (Fig. 1; circled C and D). During aligned days, both locomotor activity bouts (τ = 22 h and τ = ∼25 h) occur during the dark phase and both rest bouts during the light phase.

### Behavioral tests

For the behavioral tests, control rats were tested in either the middle of their active phase [zeitgeber time 18 (ZT18), where ZT12 is the time of lights off] or the middle of their rest phase (ZT6). Similarly, desynchronized rats were tested on aligned days in either the middle of their active phase (circled A in Fig. 1) or the middle of their rest phase (circled B in Fig. 1), or on misaligned days in either the middle of the LD22 light phase (circled C in Fig. 1) or the middle of the LD22 dark phase (circled D in Fig. 1). Rats were also weighed to the nearest 0.2 g (V41PWE1501T; Ohaus, Parsippany, NJ) once a week when their cages were changed to assess differences in body weight between groups, as body weight changes have been linked to depression (Shioiri et al., 1993).

We divided rats into three cohorts. One cohort (control: *n* = 22; desynchronized: *n* = 47) was tested in the saccharin preference test (SPT) and sexual behavior test (SBT), allowing a recovery period of at least a week between two sequential tests. A sample of rats from this cohort (control: *n* = 12; desynchronized: *n* = 19) was tested in the open field test (OFT). The second cohort (control: *n* = 16; desynchronized: *n* = 24) underwent a single FST. The third cohort (control: *n* = 9; aligned: *n* = 8) was subjected to a single OFT followed by decapitation and harvesting of brain tissue for chromatographic analysis. The OFT results from this cohort (not shown) were similar to those from cohort 1. For chromatography, we used rats only during aligned days because they showed the most pronounced differences in behavioral performance compared with control rats. All of the behavioral tests, with the exception of the SPT that was done in the rats’ home cage, were done in a ventilated chamber kept at constant temperature and humidity. Animals were transferred to the behavioral testing room in their home cages. Lighting conditions in the testing chamber were similar to those in the housing chamber at the time of the test: ∼60 lux for animals tested during the light phase and 1 lux red light for animals tested during the dark phase. The latter animals were transported to the behavioral test chamber under darkness.

### Saccharin preference test

To assess anhedonia, rats from both groups were offered two identical bottles, one filled with tap water and the other with 0.1% saccharin solution in tap water for a period of 8 days. Bottles were weighed daily to ±0.1 g to track liquid consumption of the animals over a period of 24 h, and the position of the bottles was switched daily (at 15:00, which corresponds to ZT12 for controls, and falls at different time points on the LD cycle of the desynchronized rats) to prevent possible effects of side preference in drinking. We then calculated the proportion of 0.1% sucrose consumption out of total liquid consumption. For LD24 animals, the data from all 8 days was included. During the 8-day period, each LD22 animal went through one aligned and one misaligned day; only these days were included in the analysis, and days in which the animals were neither aligned nor misaligned were not included.

### Open field test

To assess anxiety, desynchronized and control rats were placed at the lower right corner of a black square Plexiglas arena (90 × 90 × 30 cm; width × length × height). Animal movements were monitored for 10 min using a surveillance video camera (WV-BP334, Panasonic, Osaka, Japan) attached to the ceiling, 2 m above the center of the arena. We used an open-source automated movement-tracking code that uses Matlab (MathWorks, Natick, MA) as a platform (http://www.seas.upenn.edu/~molneuro/autotyping.html) to calculate the fraction of time spent in the perimeters of the arena (Patel et al., 2014), which is a measure of anxiety.

### Sexual behavior test

Sexual dysfunction is reported in 50–60% of untreated patients with depression (Kennedy et al., 1999; Bonierbale and Tignol, 2003), and sexual inquiry is recommended as an integral component of the medical evaluation (Basson and Schultz, 2007). To assess sexual function, males were placed in a transparent Plexiglas arena (40 × 60 × 40 cm; width × length × height) covered with a 5-mm layer of bedding (Bed o’Cobs, The Andersons, Maumee, OH) and were allowed 5 min to habituate to the arena before a receptive female was introduced. The behavior of the rat pair was monitored with a surveillance camera (WV-BP334, Panasonic) until the first event of ejaculation occurred or for 30 min if the male did not ejaculate. During this period, we recorded the frequency and timing of three different male behaviors: mounting, intromission, and ejaculation as previously defined by Ågmo (1997). Using this information, we calculated several standard parameters of sexual behavior: latency to ejaculation, latency to mounting, latency to intromission, and copulatory rate, as follows:

$$\text{Copulatory rate}=\frac{\text{\# mountings}+\text{\# intromissions}}{\left(\text{latency to ejaculation}-\text{latency to mounting}\right)}.$$

To induce sexual receptiveness, females were anesthetized with isoflurane by inhalation. Their ovaries were removed with a flank incision of about 1.5 cm, and another 1-cm incision to the underlying muscle tissue. The oviduct was ligated with absorbable sutures, and the ovary was then removed. The same procedure was repeated on the opposite side to remove the second ovary. The peritoneum was sutured with absorbable sutures and the skin with wound clips. Females were given at least 7 days of recovery before being used for the behavioral tests. Sexual receptiveness was induced by two injections of 20 µg of estradiol suspended in flaxseed oil 48 and 24 h before the behavioral test and an injection of 1 mg of progesterone suspended in flaxseed oil 4 h before the behavioral test.

### Forced-swim test

Rats were individually subjected to a unique 5-min FST in a separate room following Gonzalez and Aston-Jones (2008). Briefly, rats provided with a flotation aid were placed for 5 min in a tank (38.5 cm height × 30.5 cm diameter; Instech Laboratories, Plymouth Meeting, PA) with warm water (30°C). The tank was placed inside a black cylinder to reduce visual stimuli during the test. Rats were briefly handled throughout the 2 weeks before the test. The brief handling, warm water, and flotation aid were used to reduce stress as a confounding factor for the evaluation of a mood-related behavioral phenotype. Under these conditions, Gonzalez and Aston-Jones (2008) demonstrated that a single exposure to the FST can reveal a depressive behavioral phenotype in a single assay. A digital video camera over the tank recorded behaviors during the FST. Immediately after the test, each animal was removed from the water, towel-dried, and returned to its home cage. The water was changed and the cylinder cleaned between rats. Two observers blinded to the experimental conditions scored immobility (when the animal is being completely motionless, with no movements of limb, tail, or head) offline on a video monitor from videotaped images using Behavior Coding 2.18 (David Hurley, University of Washington, Seattle, WA).

### High-performance liquid chromatography (HPLC)

Immediately after the OFT (the only test these animals underwent), LD24 control rats and LD22 aligned rats were moved to an adjacent room with the same lighting conditions of the test, where they were decapitated with a guillotine. The brain was rapidly removed from the skull and placed on filter paper soaked in 0.01 m chilled PBS. A single coronal cut was made across both hemispheres of the brain at bregma + 3.7 mm, and both the left and right PFC regions (Paxinos and Watson, 1998) were dissected with fine scissors. The tissue was placed in a centrifuge tube and quickly frozen on dry ice. Less than 2 min passed from the time of decapitation to the freezing of the prelimbic and cingulate cortex tissue. The tissue was stored at –80°C until processing.

Each tissue sample was sonicated in 0.5 ml of 0.1 m perchloric acid and 2 μm ascorbic acid. A 100-μl aliquot of the sonicated material was stored at –70°C for protein determination with a Pierce BCA Protein Assay Kit (Thermo Fisher Scientific, Rockford, IL). After centrifugation of the remainder of the sonicated material at 13,000 × *g* for 15 min, the supernatant was collected and stored at –70°C for later use. After thawing, 200 μl of the supernatant was filtered through a 0.22 Millex GV syringe-driven filter and transferred to an autosampler tube before injection. Detection was performed with an ESA Coulochem II electrochemical detector (ESA Laboratories, Chelmsford, MA) with the conditioning cell set at –300 mV, electrode 1 of the analytic cell set at –90 mV, and electrode 2 of the analytic cell set at 350 mV, and a Phenomonex reverse-phase c18 Gemini column (150 × 4.6 mm, 3 µm, 110 Å; Phenomenex, Torrance, CA). The EZChrom Elite chromatography data system (Agilent Technologies, Santa Clara, CA) was used for data reduction. Norepinephrine (NE), 3-methoxy-4-hydroxyphenylglycol (MHPG), dopamine (DA), 3,4-dihydroxyphenylacetic acid (DOPAC), serotonin (5-HT), and 5-hydroxyindole acetic acid (5-HIAA) were separated and quantified. Concentrations are expressed as pg/µg protein.

### Statistical analysis

The changes in body mass and mean locomotor activity of rats from different groups were compared using repeated-measures ANOVA with rat ID as a random factor nested within treatment group. Many behavioral tests are heavily reliant on locomotor activity. To account for this confounding effect, we included mean locomotor activity in the home cage of each rat during the day of the test as a covariate. Whenever locomotor activity did not have a significant effect on behavioral performance, it was excluded from the model, and we report its effect only when found significant. Fraction of saccharin consumption was not normally distributed, and therefore we used the nonparametric Kolmogorov–Smirnov test to compare the groups and Bonferroni correction for multiple comparisons. The reported *p*-values are corrected values. Fraction of time spent in perimeter in the OFT, immobility during the FST, copulatory rate, and levels of monoamines in the PFC were all compared between groups and phase using two-way ANOVA. Because a significant number of animals did not display sexual behavior during the test, our data were strongly right skewed. For this reason, we used Kaplan–Meier survival analysis to compare differences in latency to mounting, intromission, and ejaculation between desynchronized and control rats during the dark and light phases.

We also calculated a depression score for rats that were tested on multiple behavioral tests. Because the FST was done on a different cohort of rats, it was not included. Thus, the depression score we calculated for each individual (control: *n* = 20, desynchronized: *n* = 47) was the sum of the standardized (as *z*-values obtained subtracting the mean and dividing by the standard deviation) mounting latency, standardized ejaculation latency, standardized copulatory rate, and standardized saccharin consumption. We multiplied the standardized saccharin consumption and copulatory rate by –1 to make sure that for all behavioral measures, a higher score is indicative of a higher depressive phenotype. Because the depression scores did not distribute normally, we used the Kruskal–Wallis nonparametric test to compare the scores of the three different treatment groups. Finally, we used linear regression analysis to test the relationship between performance in the different behavioral tests and the amplitude of locomotor activity rhythm on the day of the test, calculated as the difference between mean locomotor activity per hour during the dark and light phases. In the case of saccharin preference, we tested this correlation using values from a single day. Because saccharin consumption was measured every 24 h over several days, LD22 rat data are divided into aligned and misaligned at the end of the experiment by compiling days of alignment or days of misalignment for each animal. Tukey’s *post hoc* test was used for pairwise multiple comparisons when groups or the interaction were found statistically different. All statistical tests were done with R version 3.1.2 using ‘nlme,’ ‘R.matlab,’ and ‘survival’ packages.

## Results

### Body mass and locomotor activity

**Figure 2.**
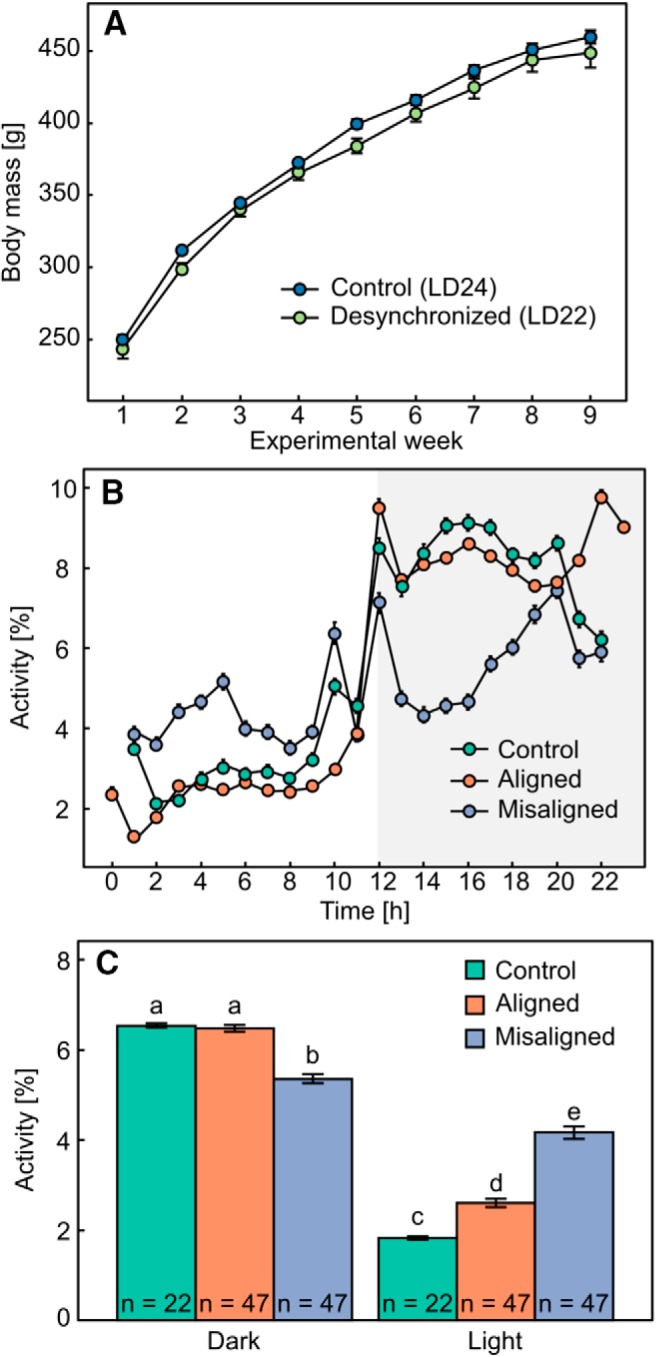
The effect of forced desynchrony on body mass and locomotor activity. ***A***, Change in mean ± SE body mass in LD24 controls (*n* = 22) and LD22 desynchronized (*n* = 47) animals throughout the experiment. ***B***, Mean ± SE locomotor activity [% of total activity during each 24- or 22-h cycle] of LD24 control rats and LD22 desynchronized rats. Shaded gray area denotes lights off. Note that data for desynchronized animals corresponds to 11 h of light and 11 h of darkness. ***C***, Mean ± SE locomotor activity per hour (total IR beam interruptions) averaged over either the light or dark phase for control rats, aligned rats, and misaligned rats. Different letters denote statistically significant differences between groups.

Body mass of control rats (372.75 ± 15.19 g, *n* = 22) did not significantly differ from that of desynchronized rats (382.17 ± 10.36 g, *n* = 47; Fig. 2*A*; *F*_1,67_ = 3.57, *p* = 0.06). As expected, the circadian rhythm of locomotor activity significantly differed between groups and phase of day (Fig. 2*B*, *C*; interaction effect *F*_2,161_ = 165.9, *p* < 0.001). Specifically, during the light phase, LD24 control rats had lower locomotor activity (*n* = 22) than LD22 rats during aligned days (*n* = 47; Tukey’s *post hoc* test: *p* < 0.001), and LD22 misaligned rats had the highest locomotor activity levels (*n* = 47; Tukey’s *post hoc* test: *p* < 0.001). During the dark phase, there was no difference between locomotor activity levels of LD24 control rats (*n* = 22) and LD22 aligned rats (*n* = 47; Tukey’s *post hoc* test: *p* = 0.9), but LD22 misaligned rats had significantly lower levels of locomotor activity (*n* = 47; Tukey’s *post hoc* test: *p* < 0.001). Finally, the amplitude of locomotor activity was significantly different between groups (*F*_2,46_ = 130.5, *p* < 0.001), where LD24 control rats had the highest amplitude of locomotor activity, and LD22 misaligned rats had the lowest amplitude compared with both LD22 aligned rats and LD24 control rats.

### Circadian forced desynchrony and behavioral performance

#### Saccharin preference test

**Figure 3.**
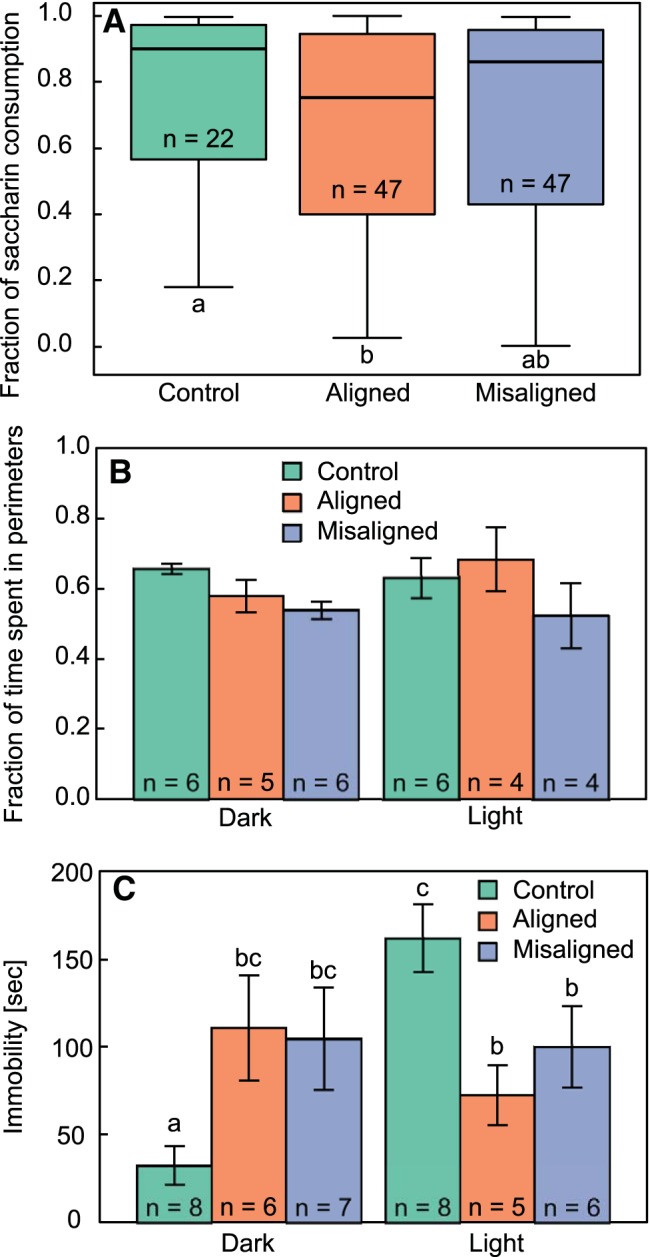
Effect of circadian forced desynchrony on behavioral manifestations of depression. ***A***, Fraction of 0.1% saccharin consumption averaged over 8 d for LD24 control, LD22 aligned, and LD22 misaligned rats. Boxes outline the lower (25%) and upper (75%) quartiles, and the line denotes the median. Error bars represent the 95% confidence intervals. Different letters denote statistically significant differences (*p* < 0.05). ***B***, Fraction of time spent in the perimeters of the open field test arena, during the light and dark phases in the same groups. ***C***, Immobility time during the 5-min FST in the light and dark phases. Different letters denote statistically significant differences (*p* < 0.05). All values represent mean ± SE.

We did not find a difference in saccharin preference throughout the 8 days of the test (*F*_1,257_ = 2.38, *p* = 0.1). This allowed us to consolidate data of LD22 desynchronized rats measured on different days, as alignment and misalignment days did not fall on the same date for each individual. LD24 control rats consumed a significantly larger fraction of 0.1% saccharin (0.76 ± 0.02, *n* = 22) than LD22 rats measured during aligned days (0.65 ± 0.03, *n* = 47; Fig. 3*A*; *D* = 0.19, *p* = 0.03), but not more than LD22 rats measured during misaligned days (0.69 ± 0.04, *n* = 47; *D* = 0.15, *p* = 0.6). Saccharin consumption did not differ between rats measured during aligned and misaligned days (Fig. 3*A*; *D* = 0.14, *p* = 0.5).

#### Open field test

No differences were found between treatment groups in the fraction of time rats spent in the perimeter of the open field test arena (Fig. 3*B*; group: *F*_2,25_ = 2.52, *p* = 0.1; group × phase interaction: *F*_2,25_ = 0.86, *p* = 0.4), and there was no difference in preference for the perimeter between the light and the dark phases (Fig. 3*B*; phase: *F*_1,25_ = 0.12, *p* = 0.7). In addition, no differences were found in the distance travelled by the rats (group: *F*_2,25_ = 1.49, *p* = 0.2, group × phase interaction: *F*_1,25_ = 0.46, *p* = 0.6), but the distance travelled by all rats during the dark phase was greater than the distance travelled by all rats during the light phase (phase: *F*_1,25_ = 18.12, *p* < 0.001).

#### Forced swim test

Immobility time during the FST was significantly different between treatment groups (Fig. 3*C*; *F*_2,34_ = 8.42, *p* = 0.001). Specifically, during the dark phase, LD24 control rats showed significantly lower immobility time than LD22 aligned (Tukey’s *post hoc* test: *p* = 0.02) and misaligned (Tukey’s *post hoc* test: *p* = 0.02) rats. During the light phase, LD24 rats had significantly higher immobility time than LD22 aligned (Tukey’s *post hoc* test: *p* = 0.01) and misaligned (Tukey’s *post hoc* test: *p* = 0.05) rats.

#### Sexual behavior test

**Figure 4.**
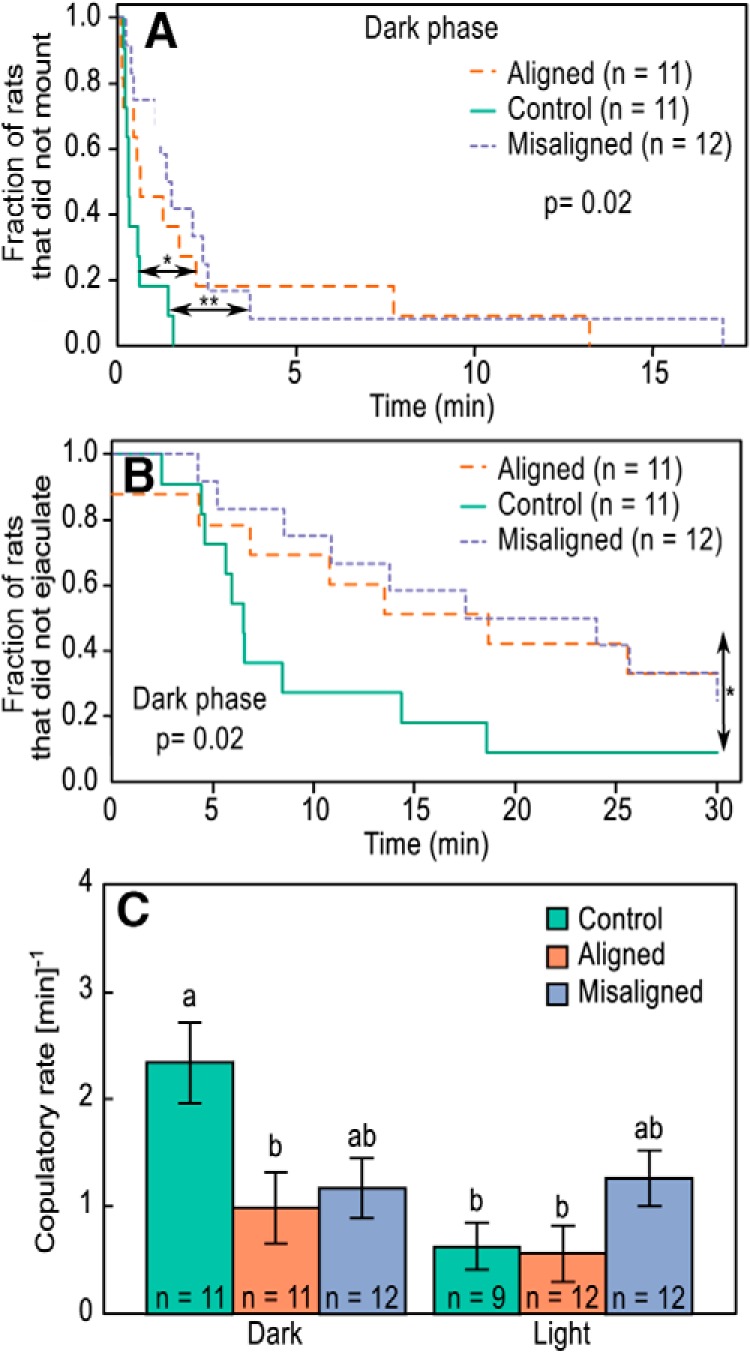
Effect of circadian forced desynchrony on sexual behavior of male rats during the dark phase. Percentage of LD24 control and LD22 desynchronized male rats that did not mount (***A***) and did not ejaculate (***B***) as a function of the time lapsed from the introduction of a receptive female. *p < 0.05, **p < 0.01. Log-rank values were calculated based on a Kaplan–Meier survival analysis. ***C***, Mean ± SE copulatory rate in LD24 control and LD22 desynchronized males. Copulatory rate was calculated as the sum of mounting and intromission events divided by the time lapsed from the first mounting event until the first ejaculation event. Different letters denote statistical differences between groups (*p* < 0.05).

Circadian forced desynchrony had an effect on sexual behavior. During the dark phase (Fig. 4*A*; χ^2^ = 7.83, *p* = 0.02) LD24 control rats (*n* = 11) had a shorter mounting latency than both aligned (*n* = 11; Tukey’s *post hoc* test: *p* = 0.01) and misaligned (*n* = 12; Tukey’s *post hoc* test: *p* = 0.003) rats, but no such differences were found in the light phase (χ^2^ = 2.46, *p* = 0.3). Similarly, a difference in ejaculation latency was found during the dark phase (Fig. 4*B*; χ^2^ = 7.89, *p* = 0.02), with LD24 control rats (*n* = 11) having shorter ejaculation latency than LD22 aligned (*n* = 11; Tukey’s *post hoc* test: *p* = 0.02) but not misaligned (*n* = 12; Tukey’s *post hoc* test: *p* = 0.06) rats. When we included mean locomotor activity as a covariate, we found that it had a significant effect on ejaculation latency (*F*_1,56_ = 4.20, *p* = 0.05), but this effect did not alter the results because the effect of group remained significant (*F*_2,56_ = 4.85, *p* = 0.01). No differences in ejaculation latency were found during the light phase (χ^2^ = 3.70, *p* = 0.2). Furthermore, no difference was found in intromission latency during the light (χ^2^ = 2.79, *p* = 0.3) or dark (χ^2^ = 2.11, *p* = 0.3) phases. Finally, LD22 aligned rats had lower copulatory rates than LD24 control rats during the dark phase (Fig. 4*C*; *F*_2,62_ = 4.71, *p* = 0.01), whereas misaligned rats did not differ from aligned (Tukey’s *post hoc* test: *p* = 1.0) or LD24 control (Tukey’s *post hoc* test: *p* = 0.07) rats. Mean locomotor activity had a significant effect on copulatory rate (*F*_1,56_ = 9.65, *p* = 0.003), but even when this effect was included in the model, we found a significant effect of group (*F*_2,56_ = 3.63, *p* = 0.03).

Because females were tested at different time points during their 24-h cycle, we included the female ZT as a covariate in our analysis. We found no effect of the female ZT on either ejaculation latency (*F*_1,66_ = 2.39, *p* = 0.1) or mounting latency (*F*_1,66_ = 1.51, *p* = 0.2). We did find a significant effect of the female ZT on copulatory rate (*F*_1,66_ = 3.99, *p* = 0.05). However, when accounting for this effect, we still found a significant difference between groups (group: *F*_2,56_ = 3.49, *p* = 0.04; group × phase interaction: *F*_2,56_ = 4.30, *p* = 0.02), with LD24 control rats showing higher copulatory rate during the dark phase compared with LD22 aligned rats (Tukey’s *post hoc* test: *p* = 0.05), but not compared with LD22 misaligned rats (Tukey’s *post hoc* test: *p* = 0.1). Copulatory rate of LD22 misaligned rats was also not statistically different from that of LD22 aligned rats (Tukey’s *post hoc* test: *p* = 0.99). Thus, the effects were identical to those we report above.

#### Depression score

**Figure 5.**
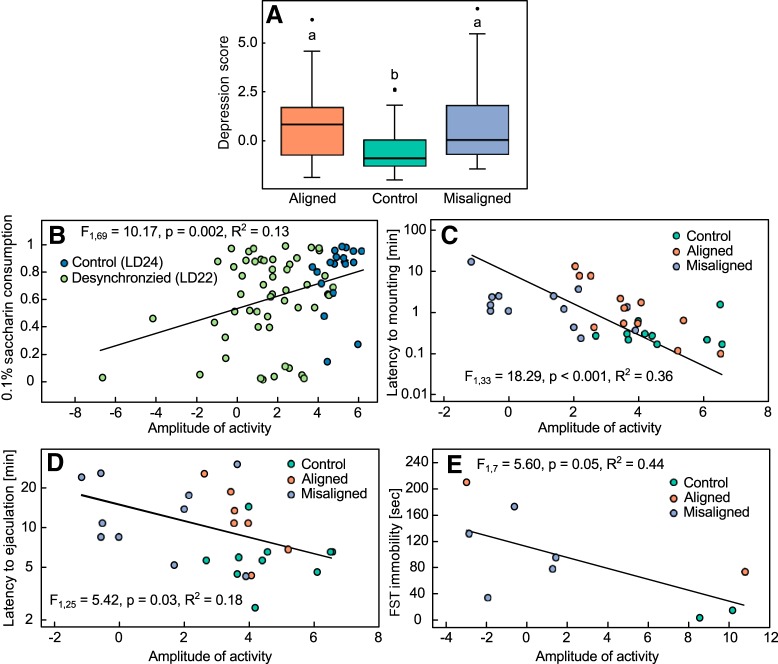
The effect of circadian forced desynchrony on behavioral performance. ***A***, A depression score (higher values indicate a depressive phenotype) for LD24 control, LD22 aligned, and LD22 misaligned rats. Boxes outline the lower (25%) and upper (75%) quartiles, and the line denotes the median. Error bars represent the 95% confidence intervals, and the black circles are outliers. Different letters denote statistically significant differences (*p* < 0.05). The amplitude of circadian locomotor activity predicts the severity of behavioral manifestations of depression. ***B***, Fraction of 0.1% saccharin consumption over a 24-h period in LD24 control and LD22 desynchronized animals as a function of the amplitude of locomotor activity on the day of the test. Latency to mounting (***C***) and ejaculation (***D***) during the dark phase as a function of the amplitude of locomotor activity on the day of the test. ***E***, Immobility in the FST during the dark phase as a function of the amplitude of locomotor activity on the day of the test, calculated as the difference between the mean activity per hour during the dark phase and the mean activity per hour during the light phase.

We found a significant difference between scores of different groups (Fig. 5*A*; χ^2^_(2)_ = 7.63, *p* = 0.02). Specifically, the depression score of control rats (–0.43 ± 0.27) was significantly lower than that of LD22 aligned (0.89 ± 0.45; *post hoc* test: *p* = 0.03) as well as LD22 misaligned (0.84 ± 0.42; *post hoc* test: *p* = 0.03) rats.

#### Correlations between amplitude of locomotor activity and performance in the behavioral tests

The fraction of 0.1% saccharin consumption over a period of 24 h was positively correlated with the amplitude of locomotor activity during the day of the test (Fig. 5*B*; *F*_1,69_ = 10.17, *p* = 0.002, *R* ^2^ = 0.13). Additionally, both latency to mounting (Fig. 5*C*; *F*_1,33_ = 18.29, *p* < 0.001, *R* ^2^ = 0.36) and latency to ejaculation (Fig. 5*D*; *F*_1,25_ = 6.29, *p* = 0.02, *R* ^2^ = 0.20) in the dark phase were negatively correlated with the amplitude of locomotor activity during the day of the test. Finally, we found a significant positive correlation between immobility in the FST and the amplitude of locomotor activity during the day of the test (Fig. 5*E*; *F*_1,7_ = 5.60, *p* = 0.05, *R* ^2^ = 0.44).

### Circadian forced desynchrony and prefrontal cortex monoamine levels and turnover

**Figure 6.**
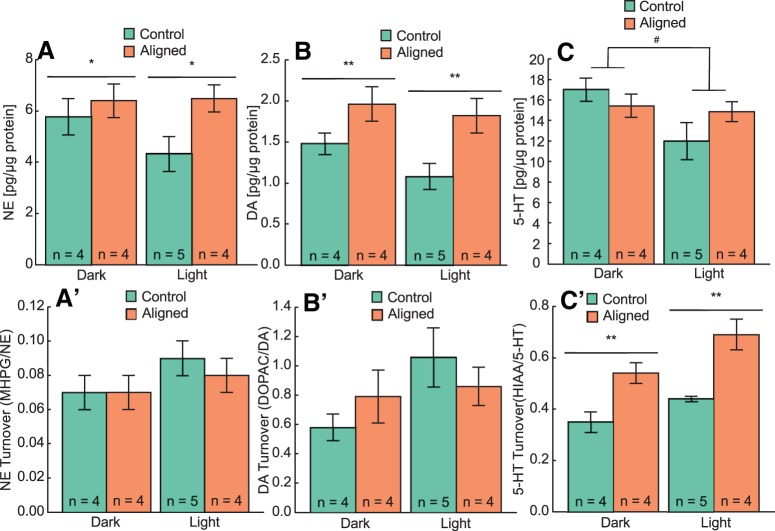
Forced desynchronized rats show a significant increase of levels of NE and DA and of 5-HT turnover in the prefrontal cortex. ***A–C***, Mean ± SE levels of each monoamine. ***A′–C′***; mean ± SE turnover for each monoamine. *p < 0.05, **p < 0.01 (group effect); #*p* = 0.05 (phase effect). See Table 1 for statistics.

We analyzed the amount of NE, DA, and 5-HT and their respective metabolites in the PFC at the same phases in which behavioral tests were performed in LD24 and LD22 aligned animals (Fig. 6). Table 1 shows monoamine and metabolite levels, their calculated turnover, and statistical results. NE (Fig. 6*A*; F_1,28_ = 4.49, *p* = 0.04) and DA (Fig. 6*B*; F_1,28_ = 10.21, *p* = 0.003) levels were higher in LD22 desynchronized rats compared with LD 24 controls (25% and 48%, respectively), and neither group showed a circadian oscillation in NE (Fig. 6*A*; *F*_1,28_ = 1.10, *p* = 0.3) or DA (Fig. 6*B*; *F*_1,28_ = 2.00, *p* = 0.2) levels. 5-HT levels were higher during the dark than during the light phase in both groups (Fig. 6*C*; *F*_1,15_ = 4.60, *p* = 0.05), and there were no differences in 5-HT levels between LD24 controls and LD22 desynchronized rats (Fig. 6*C*; *F*_1,15_ = 0.24, *p* = 0.6). Finally, there were no differences in turnover rates of NE (Fig. 6*A′* ; *F*_1,28_ = 0.89, *p* = 0.4) or DA (Fig. 6*B′* ; *F*_1,28_ = 0.0006, *p* = 0.98) between groups, but the turnover rate of 5-HT was significantly higher in LD22 desynchronized rats than in LD 24 controls (Fig. 6*C′* ; *F*_1,15_ = 12.80, *p* = 0.003).

**Table 1.** Monoamine levels in PFC of LD24 controls and LD22 aligned desynchronized rats.

Substance	LD24		LD22		Two-way ANOVA		
	Light	Dark	Light	Dark	Group	Phase	Interaction
Monoamine (pg/μg protein)							
NE	4.33 ± 0.67	5.78 ± 0.70	6.48 ± 0.53	6.39 ± 0.66	***F*_1,28_ = 4.49; *p* = 0.04**	*F*_1,28_ = 1.10; *p* = 0.3	*F*_1,28_ = 1.39; *p* = 0.2
DA	1.08 ± 0.16	1.48 ± 0.13	1.82 ± 0.21	1.96 ± 0.21	***F*_1,28_ = 10.21; *p* = 0.003**	*F*_1,28_ = 2.00; *p* = 0.2	*F*_1,28_ = 0.44; *p* = 0.5
5-HT	12.00 ± 1.79	17.00 ± 1.13	14.86 ± 0.96	15.40 ± 1.13	*F*_1,15_ = 0.24; *p* = 0.6	***F*_1,15_ = 4.60; *p* = 0.05**	*F*_1,15_ = 2.97; *p*=0.1
Metabolite (pg/μg protein)							
MHPG	0.39 ± 0.06	0.37 ± 0.03	0.48 ± 0.03	0.41 ± 0.04	*F*_1,28_ = 3.03; *p* = 0.09	*F*_1,28_ = 1.17; *p* = 0.3	*F*_1,28_ = 0.38; *p*=0.5
DOPAC	1.12 ± 0.23	0.89 ± 0.14	1.41 ± 0.14	1.19 ± 0.30	*F*_1,27_ = 2.34; *p* = 0.1	*F*_1,27_ = 1.43; *p* = 0.2	*F*_1,27_ = 0.01; *p* = 0.9
HIAA	5.25 ± 0.15	5.82 ± 0.50	10.26 ± 1.24	8.22 ± 0.49	***F*_1,15_ = 10.52; *p* = 0.006**	*F*_1,15_ = 0.41; *p* = 0.5	*F*_1,15_ = 1.30; *p* = 0.3
Turnover							
NE (MHPG/NE)	0.09 ± 0.01	0.07 0.01	0.08 ± 0.01	0.07 ± 0.01	*F*_1,28_ = 0.89; *p* = 0.4	*F*_1,28_ = 3.36; *p* = 0.08	*F*_1,28_ = 1.53; *p* = 0.2
DA (DOPAC/DA)	1.06 ± 0.20	0.58 ± 0.09	0.86 ± 0.13	0.79 ± 0.18	*F*_1,28_ = 0.0006; *p* = 0.98	*F*_1,28_ = 3.12; *p* = 0.09	*F*_1,28_ = 0.20; *p* = 0.2
5HT (HIAA/5HT)	0.44 ± 0.01	0.35 ± 0.04	0.69 ± 0.06	0.54 ± 0.04	***F*_1,15_ = 12.80; *p* = 0.003**	*F*_1,15_ = 3.96; *p* = 0.07	*F*_1,15_ = 0.22; *p* = 0.6

Levels (pg/µg of protein) of monoamines and their metabolites are shown as mean ± SE. Turnover is shown as the ratio of each metabolite over each respective monoamine. The main effects of the two-way ANOVA are shown. Values in bold are significant.

## Discussion

The link between affective disorders and disruptions of the circadian system has been recognized for decades (Wulff et al., 2010; McClung, 2013). Nevertheless, it is unclear whether circadian disorders may represent a comorbidity of mental disease or, alternatively, challenges to circadian organization may be among the prevalent factors that increase susceptibility to mood disorders. Our results show that long-term desynchronization of the circadian system in a neurally, genetically, and pharmacologically intact mammal leads to depressive-like behavior, as indicated by increased immobility in the FST and decreased hedonic behavior in the SPT and SBT (Figs. 3 and 4). Moreover, we found that the severity of the circadian desynchronization, as estimated by the amplitude of locomotor activity, was correlated with performance in the behavioral tests (Fig. 5). Specifically, the lower the amplitude of locomotor activity rhythm, the stronger the depressive phenotype was on the behavioral tests. These results suggest that internal circadian desynchronization contributes to the pathogenesis of affective disorders.

The manipulation of the LD cycle in the forced desynchronized rat leads to the desynchronization of circadian rhythms, including the rhythms of core body temperature, release of corticosterone and melatonin, and REM sleep (de la Iglesia et al., 2004; Cambras et al., 2007; Lee et al., 2009; Schwartz et al., 2009; Wotus et al., 2013). The internal misalignment of these circadian rhythms is characteristic of depression in humans as well (Souêtre et al., 1989; Riemann et al., 2001; Lewy, 2009). This misalignment is in agreement with the pattern of clock gene expression in extra-SCN neural circadian oscillators in postmortem tissue from human patients with depression and in animal models of depression (Li et al., 2013; Landgraf et al., 2015; Logan et al., 2015; Savalli et al., 2015). However, depression in the animal models used in these studies was induced by aversive methods, and the causality between desynchronization and the manifestation of depression could not be established. In the forced desynchronized rat, the simple manipulation of the LD cycle causes the predictable and stable desynchronization of neuronal oscillators within the SCN in the absence of any invasive intervention, and this in turn leads to a depressive behavioral phenotype.

For the present study, we used a single-exposure FST that has been previously validated in an animal model of light deprivation–induced depression. Rats kept for 6 weeks in constant darkness showed increased immobility in the first 5 min of exposure to the test compared with controls, and this behavioral deficit was reversed by desipramine (Gonzalez and Aston-Jones, 2008). Similarly, desynchronized rats during the dark phase in our study showed a spontaneous despair behavior revealed by the increased immobility during the 5-min FST, an expected outcome if circadian desynchrony predisposes individuals to a behavioral depressive phenotype. Importantly, the FST depressive phenotype observed in aligned LD22 animals during the dark phase, compared with LD24 animals during the same phase, cannot be attributed to reduced locomotor activity because the activity in the home cage for these groups did not differ (Fig. 2*B*, *C*). We argue that this reduced response—increased immobility—to helplessness during a time of the day at which the animals are expected to be more active represents a phenotypic manifestation of depression.

Control rats kept under LD24, showed a daily (likely circadian) modulation of performance in the FST, manifested as less immobility during the dark phase. This modulation has been largely ignored in studies using behavioral markers of depression, which typically perform the tests during the rest (light) phase of the animals. Indeed, Tataroğlu et al. (2004) have shown that bilateral lesion of the SCN in rats leads to reduced behavioral despair, and suggested that the lesion had an ameliorative effect on the strategy to respond to the stress of the FST. Because the animals in that study were tested during the light phase, an alternative explanation that we favor for this result is that the lesions do not reduce despair but instead eliminate the circadian modulation of the FST performance.

In contrast to LD24 rats, LD22 animals did not show similar circadian modulation of FST performance, as evident from the lack of differences in levels of immobility between the dark and light phases. Specifically, rats tested during both aligned and misaligned days showed higher immobility during the dark phase and lower immobility during the light phase compared to controls. Because mood was shown to oscillate daily in humans from different cultures around the globe (Golder and Macy, 2011) and a blunted rhythm of mood during the 24 h day was found in depressive patients (Boivin, 2000; Wirz-Justice, 2006), we suggest that LD22 aligned rats show attenuated rhythm of immobility in the FST, characterized by respectively lower and higher immobility during the light and dark phases than controls, and no difference in levels of immobility between the two phases.

Strikingly, we found that LD22 desynchronized rats displayed lower levels of immobility during the light phase than LD24 control rats (Fig. 3*C*). We do not think that this is an indication of manic behavior for two reasons. First, LD22 rats consistently display anhedonia in the other behavioral tests. Second, because misaligned animals are tested in conflicting phases—subjective day during the dark phase or subjective night during the light phase—their immobility in the FST could reflect the opposing effects of light and the circadian system on escape performance rather than the depressive state of the animal. In line with this interpretation, immobility in LD22 misaligned animals during the light phase was higher than immobility in LD24 animals during the dark phase but lower than immobility in in LD24 animals during the light phase.

Our study is the first to show that internal circadian desynchronization leads to sexual dysfunction. We found that circadian desynchronization affects both sexual motivation, estimated by the latency to mount (Pfaus et al., 1990), and sexual function, estimated by the latency to and occurrence of ejaculation (Ågmo, 1997). Moreover, the severity of the circadian disturbance, i.e., the reduction in amplitude of locomotor activity during the day of the test, was correlated with both measures (Fig. 5*C*, *D*), suggesting that the impairment of sexual behavior is linked to the extent of circadian disruption. The prevalence of sexual dysfunction in depressive patients not treated with antidepressants is estimated to be 50–60%, but this value is likely to be an underestimation because sexual inquiry is often not included in the medical assessment (Kennedy et al., 1999; Bonierbale and Tignol, 2003; Basson and Schultz, 2007). Our results suggest that circadian desynchronization may be a contributing factor to both negative affect and sexual dysfunction. Of note, sexual dysfunction has not been previously tested in animal models of depression, and our results show that it represents a sensitive behavioral outcome to assess the depressive phenotype of an animal, one that is more meaningful than other metrics for both its ecophysiological and translational value. Specifically, the FST has been criticized as a behavioral assessment for depression because (1) it is heavily dependent on locomotor activity performance (Slattery and Cryan, 2012) and (2) as a method to induce depression, it represents an unusually stressful situation that bears little relationship to depression triggers in humans (Holmes, 2003). In the present study, we did not use the FST to induce depression but rather as a probe test to assess a preexisting depressive state. Nevertheless, it still can be argued that a test in which an individual has to swim after being placed in an unescapable pool of water is unlike most stressful situations that lead to a manifestation of depression in humans. In this context, we propose that assessing sexual performance may represent a more sensitive and meaningful testing approach in animal models of depression.

The depressive-like phenotype we found was independent of levels of anxiety, as evident from the lack of differences between desynchronized and control preference for the perimeter of the arena in the OFT (Fig. 3*B*). Although there is a strong comorbidity between anxiety disorders and depression, they are still considered independent disorders. This suggests that the desynchronized rat may serve as a model for exploring depressive behavior independently of anxiety, although further tests of anxiety level are required to confirm this finding.

The correlation between abnormal light exposure, disrupted rhythms, and mood disorders suggests that photic stimulation of the circadian system through the RHT is critical for the maintenance of mood. This mechanism could explain seasonal affective disorder (SAD), a form of depression linked to the short winter photoperiod that is responsive to bright light therapy (Golden et al., 2005). The circadian basis of light therapy–induced improvement in mood in SAD patients is supported by the fact that light appears to be more beneficial when applied at a specific circadian phase: for some patients morning treatment is efficacious, and for others, evening treatment (Terman and Terman, 2005). Furthermore, light therapy is beneficial not only in SAD but in virtually all forms of depression, suggesting that circadian pathologies could contribute to the etiology of depression in general (Pail et al., 2011). Remarkably, nocturnal rats present an anatomical and behavioral depressive phenotype after weeks of exposure to total darkness. The absence of light alone reduces the amplitude of the SCN-dependent sleep-wake circadian rhythm and generates neural damage in monoamine brain systems that are SCN-transynaptic targets, in association with a depressive behavioral phenotype (Gonzalez and Aston-Jones, 2006, 2008). Interestingly, Dulcis et al. (2013) showed that the type of neurotransmitter released from nuclei receiving direct SCN input is dependent on photoperiod length, and that the alteration in neurotransmitter release is associated with a depressive-like behavior in mice. Forced desynchronized rats, however, are not exposed to different amounts of light, as the LD22 schedule results in the same proportion of light and dark as an LD24 schedule. Thus, the depressive behavioral phenotype of forced desynchronized rats likely emerges from the stable desynchrony between the vl- and dmSCN. This appears to differ from the depressive phenotype that emerges in mice exposed to aberrant LD cycles of 7 h (3.5L:3.5D), in which the SCN appears not to be affected by the schedule and the phenotype has been proposed to be mediated by retinal innervation of limbic regions (LeGates et al., 2012, 2014).

The monoamine hypothesis of depression postulates that the pathogenesis of depression involves impairment of 5-HT, NE, and DA systems in the central nervous system (Delgado, 2000; Hirschfeld, 2000), and there is evidence that these abnormalities are localized to the PFC and the limbic system (Drevets, 1999). Our desynchrony protocol leads to an imbalance in the regulation of monoamine levels and their release in the PFC, which is manifested as higher levels of NE and DA and higher 5-HT turnover rate (Fig. 6). The role of DA in depression is still not well understood and is challenged by the paradoxical antidepressant effect of both agonists and antagonists of DA (Dailly et al., 2004). In stressed rats, there is increased release of NE and DA in the PFC, and chronic mild stress causes an increase in the magnitude of this response (Finlay et al., 1995; Di Chiara et al., 1999). It is therefore possible that the higher levels of NE and DA we found in the PFC of LD22 desynchronized rats result from stress sensitization of these individuals, as brain tissue of these rats was collected immediately after an OFT. In contrast, we found unchanged 5-HT levels combined with higher 5-HT turnover rate in LD22 desynchronized rats, suggesting that these animals have a lower availability of 5-HT, which could account for their depressive like behavior. This is also consistent with decreased availability of 5-HT in patients with SAD and the 24% decrease in serotonergic axons found in the PFC of suicide victims (Austin et al., 2002). Our findings provide a direct link between stable circadian desynchronization and an imbalance in monoamine regulation in the PFC. Obviously, the PFC is not the only brain region involved in the modulation of motivational states, and changes in other transmitter systems, as well as in intracellular regulatory pathways within or outside the PFC, could underlie the expression of depressive behaviors in the forced desynchronized rat.

In summary, our study shows that the stable forced desynchrony of circadian rhythms leads to the manifestation of a depressive-like phenotype. This phenotype likely emerges from the desynchronization of neuronal oscillators within the SCN and is associated with the severity of circadian desynchronization and an imbalance in monoamine metabolism within the PFC. Taken together, our findings provide direct support for a circadian desynchronization etiology of depressive behavior. Hence, the forced desynchronized rat could represent a unique neurologically, pharmacologically, and genetically intact animal model to study the neural basis of affective disorders and its relationship with internal circadian misalignment.

*Note added in proof:* The affiliation of several authors were incorrect and funding sources not included on the article published on-line December 5, 2016, as an Early Release. The affiliations list has since been corrected and the funding sources “Geriatric Research, Education and Clinical Center, and the Research and Development Service of the VA Puget Sound Health Care System” were added.

## Synthesis

Reviewing Editor: Rae Silver, Columbia University Decisions are customarily a result of the Reviewing Editor and the peer reviewers coming together and discussing their recommendations until a consensus is reached. When revisions are invited, a fact-based synthesis statement explaining their decision and outlining what is needed to prepare a revision will be listed below. The following reviewer(s) agreed to reveal their identity: David Welsh.

Synthesis of reviews

In the present study, the authors ask whether SCN desynchrony is sufficient to cause depressed behavior, using a rat model in which previous work shows that the two major subregions of SCN are stably desynchronized by a 22 h light/dark cycle (LD22). The authors suggest that compared to LD24 controls, desynchronized rats show more depression-like behavior: reduced saccharin preference, impaired male sexual behavior, and increased immobility in the forced swim test (FST). In the prefrontal cortex (by HPLC), these rats show higher levels of norepinephrine and dopamine, and faster serotonin turnover.

Numerous questions about the methods and interpretation arose in the review. A major problem is that the control animals seem more depressed than the desynchronized animals in some tests at some time points, and not more "depressed" overall. Specifically, for the two mood-related behaviors measured separately in light vs. dark phases (FST, sexual function), control LD24 rats appear more depressed in the light (their sleep phase) than in the dark, and LD22 rats are intermediate, i.e. no more depressed than LD24 rats in the light phase. In the FST, "LD22 desynchronized rats displayed lower level of immobility during the light phase than LD24 control rats" (line 378). Do these tests reveal in LD22 rats a true stable "depression-like" state, or only a damped daily cycle of mood or sleepiness? The authors should discuss these alternative interpretations as this is a major factor in interpreting the results.

-The assessment of the basal differences in overall activity levels (Figure 1) show very large differences between controls and LD22 rats, and thus it is difficult to use tests that are very heavily reliant on activity when activity is strongly impacted.

- The protocol used may produce acute effects of the light cycle or change in light exposure during behavioral testing. That is, moving rats from darkness into light and vice versa for different tests complicates the effects of the LD22 cycle.

- How the SPT was undertaken and results quantified. The saccharine was provided daily for 8d, and amount consumed was calculated. But the reported statistics seem to be based upon only one day. Or do the authors report the average on aligned vs. non-aligned days? Do they then compare the controls over the same days, or do they just compare to the entire 8d average? There is much published evidence that over several days of exposure, the amount of saccharine consumed changes. Was there any change over days? What is the structure of the statistical analyses, as it seems that the LD22 rats were measured twice (aligned vs. non-aligned), so does this artificially increase the overall N in the three-way comparison?

-For the OFT, more information must be provided. What was the light level? The simple measure of time on perimeter is insufficient to determine an anxiety-like phenotype. What was their overall activity in the novel arena? How many center crossings were observed? Was any stereotyped behavior observed? Were there changes in rearing behavior? These are all important measures in assessing anxiety-like behavior, and made more important given the large difference in basal activity reported in Figure 1.

-The FST results also require clarification. What are the light levels (indicating "similar" LD cycles in home and test cage is not sufficient)? Second, the Aston-Jones 2008 paper (which was cited) used many more measures of depressive like behaviors, including climbing, pawing, swimming, and immobility. They also measured diving behaviors. These behaviors were further assessed over different time blocks during the test, showing that there were differences based upon time within the test as well. The present study only shows one measure of behavior (immobility) which may not be sufficient to declare a "depressive" phenotype. Such caveats must be noted. Additionally, the behavior seems to be modulated by time of day, showing that rather than having increased depression-like behavior, during the light phase the disrupted rats are actually less depressive-like! The argument that the authors make to attempt to address this point (e.g. a blunting of rhythms of mood) is grasping at straws, particularly with only one behavior (immobility) being reported. Finally, the FST experiment uses a small N for such behavioral studies (n=4-6/group) (the Aston-Jones study used n=19/group).

While there is a link between depression and sexual drive (and general motivational deficits), the inclusion of the reproductive data seems problematic. How do the authors control for lower activity of the LD22 rats contributing to the shift in copulatory rate? This is evidenced in the light phase, when the LD22 rats have a somewhat higher rate compared to controls, which goes along with the general difference in overall activity (i.e. they are more active than controls at this time, and also show more copulatory activity than controls at this time). Indicate the LD cycle of the female. What phase were the females in, and would the LD22 males have performed better in the light phase had the females been in their dark phase?

It will be interesting to calculate the relationship among the various behavioral measures within individuals as a collective index of depression.

The methods list a total of 22 controls and 47 LD22 for the SPT, OFT, and SBT, and that the rats were run through all of the tests, with a one week recovery time between each. In Figure 3, the N in the SPT is correct, but the aligned and misaligned rats are counted twice (i.e. rats are measured when they are aligned and also when misaligned). However, in the OFT, there are only 12 total controls, and 19 total LD22, but it isn't clear that it is different rats that are tested in the dark and light, and also if they are also retested when aligned or misaligned as in the SPT. A similar problem is observed in the sexual behavior test, where a total of 20 controls and 48 LD22. This does not add up, yet the methods suggest all rats were measured on all tests.

Why study PFC in the HPLC experiment, given the intended focus on depressive-like behaviors. While the PFC does show changes in MA levels in depression, but MA levels in areas such as the LC and hippocampus seem more solidly connected to depressive-like behaviors. Additional brain areas would have greatly strengthened this argument. Aside from this, the results are puzzling. It is my understanding that the monoamine hypothesis of depression would predict lower levels of MA in the PFC, but unless I am misreading the figures, the results in LD22 show higher levels of NE and DA, with no change in 5HT. The authors' attempts to address this are unclear.

Minor Comments:

1) Roybal et al. (2007) used Clock-d19 mutants and Landgraf et al. (2016) used SCN-specific Bmal1 knockdown, neither of which is "KO" (lines 71, 74). If using "KO", please define abbreviation on first use.

2) Change all occurrences of "22 light-dark" to "22 h light-dark" (e.g., lines 10 and 30), "24 LD" to "24 h LD" (e.g., line 31). Remove spaces in all occurrences of X˚C, e.g. change "4 ˚C" to "4˚C".

3) Define "12:12 LD cycle" (line 103) and "11:11 LD cycle", e.g. "12:12 LD cycle (12 h light, 12 h dark)".

4) Landgraf et al. did not use chronic stress (line 358). But what's wrong with using chronic stress?

5) Change "phenotype" to "phenotypes" (lines 17, 32), "would" to "may" (21), "nucleus to" to "nucleus, to" (31), "single-cells" to "single cells" (39), "oscillated with" to "had" (120), "toejaculation" to "to ejaculation" (184), "tomounting" to "to mounting" (184), "data was" to "data were" (247), "rats are divided in" to "rat data are divided into" (255), "differ than" to "differ from" (262), "control rat" to "control rats" (276), "in the PFC and their respective metabolites" to "and their respective metabolites in the PFC" (320), "each monoamine" to "monoamine" (322), "higher" to "were higher" (324), "a depressive-like" to "depressive-like" (341), "lower level" to "a lower level" (378), "attenuated rhythm" to "an attenuated rhythm" (384), "depression, our" to "depression. Our" (402), "a light" to "light" (425), "a depressive-like behavior" to "depressive-like behavior" (431), "states and" to "states, and" (460), "shading indicate" to "shading indicates" (636), "area denote" to "area denotes" (651), "LD222" to "LD22" (671).

6) Cite Sokolove & Bushell, 1978 (line 115). Cite and discuss the SCN lesion study by Tataroglu (2004).

7) Why use only male rats?
